# Research on the Perceived Quality of Virtual Reality Headsets in Human–Computer Interaction

**DOI:** 10.3390/s23156824

**Published:** 2023-07-31

**Authors:** Yongzhong Yang, Linling Zhong, Shihui Li, Aixian Yu

**Affiliations:** Department of Business Administration, Business School, Sichuan University, Chengdu 610207, China

**Keywords:** sensor technology, human–computer interaction, VR headsets, consumption big data, perceived quality, perceived quality framework, PQAIR

## Abstract

The progress of commercial VR headsets largely depends on the progress of sensor technology, the iteration of which often means longer research and development cycles, and also higher costs. With the continuous maturity and increasing competition of VR headsets, designers need to create a balance among user needs, technologies, and costs to achieve commercial competition advantages. To make accurate judgments, consumer feedback and opinions are particularly important. Due to the increasing maturity in the technology of commercial VR headsets in recent years, the cost has been continuously decreasing, and potential consumers have gradually increased. With the increase in consumer demand for virtual reality headsets, it is particularly important to establish a perceptual quality evaluation system. The relationship between consumer perception and product quality determined by evaluations of experience is improving. Using the research method implemented in this work, through semi-structured interviews and big data analysis of VR headset consumption, the perceptual quality elements of VR headsets are proposed, and the order of importance of perceptual quality attributes is determined by questionnaire surveys, quantitative analysis, and verification. In this study, the perceptual quality elements, including technical perceptual quality (TPQ) and value perceptual quality (VPQ), of 14 types of VR headsets were obtained, and the importance ranking of the VR headsets’ perceptual quality attributes was constructed. In theory, this study enriches the research on VR headsets. In practice, this study provides better guidance and suggestions for designing and producing VR headsets so that producers can better understand which sensor technology has met the needs of consumers, and which sensor technology still has room for improvement.

## 1. Introduction

With the rise of emerging technologies such as 5G, artificial intelligence, and virtual reality, the development of intelligent wearable devices has been accelerated. Human–computer interaction has been widely used in people’s daily lives. It brings great convenience while profoundly changing people’s work, entertainment, and lifestyles. The relationship between humans and machines has entered the era of human–computer interaction; rather than constituting a single way for human beings to obtain information from computers, we are gradually realizing the development of this relationship into a two-way accurate transmission of information [1]. VR headsets can provide people with a wealth of entertainment and professional applications and are an important device to help people participate in the era of human–computer interaction. The current, rapid development of computer technology, image processing, multimedia technology, sensors, and network technology has brought new possibilities for virtual reality technology. People can interact with the virtual world with experiences that seem more real than they were previously [2]. When people wear VR headsets, they can be in a three-dimensional world, giving them a new sense of reality [3]. To achieve an immersive effect, the application of various sensor technologies is very important. In recent years, research into VR headsets has focused more on the optimization of various sensing technologies such as distance sensors, tactile sensors, and force sensors. These studies have verified the necessity and feasibility of technological progress from a technical point of view. However, few studies have discussed and verified the rationality of the optimization of various sensing technologies from the perspective of consumers. In the face of increasingly fierce competition in the market, this paper believes that researchers and producers need to pay more attention to consumers’ opinions and feedback while studying the iteration of sensor technology.

According to TrendForce, a data website, global VR headset equipment shipments reached about 8.58 million units in 2022, and it is predicted that global VR headset equipment shipments will soar to 10.35 million units in 2023, an increase of 20.6% over the same period in the prior year. Based on the theory of perceptual quality, this study aims to determine the importance ranking of the perceptual quality attributes of VR headsets, and provide a reference for manufacturers to design and manufacture VR headsets from consumers’ perceived quality.

## 2. Literature Review

### 2.1. Research on VR Headsets

At present, the research on VR headsets can be roughly divided into two directions: one is the technologies used and the other is the applications.

In relation to VR headset technologies, VR headsets are the integration products of software and hardware technologies. For example, they integrate computer graphics, digital image processing, multimedia technology, sensor technology, artificial intelligence and other software technologies [4]. Through VR headsets, users can interact with computer-generated visual outputs, such as visualization options and animations, which float in front of the user. Many scholars have studied the visual system of VR headsets, because the visual system is the core element [5]. For example, Lu discusses how to improve the VR eye tracking system to achieve better tracking results [6]. Xu discusses how to improve the performance of tactile sensors and force sensors for wearable devices, including VR headsets [7]. Dempsey and Rossz study the software and hardware technologies of VR headsets as a whole by evaluating the HTC Vive and Razer OSVR HDK, respectively [8,9].

The research on the applications of VR headsets is another direction of study in the academic world, which includes the analysis of VR technologies’ current applications as well as its prospects in these fields, such as medical care, games, education, sports, fashion, tourism, and so on. In the medical field, for instance, VR technology has been used as a treatment for PTSD [10] and for rehabilitation training after a stroke [11]. Three-dimensional gaming is an important field of VR applications, and the evolution of the game also plays a certain role in promoting the development of VR technology. More games are evolving in the direction of VR technology [12]. In the field of education, VR headsets have the advantage of breaking the limitations of time, space, and resources. Through human–computer interaction and immersive experience, a student’s imagination is stimulated, and teaching is realized by playing. Furthermore, VR headsets can avoid some experimental operation risks [13]. VR headsets can also help athletes to carry out dynamic balance and reaction time [14]. Chen combines VR technology with MTM (MadetoMeasure) which allows people to try on clothes at home [15]. If you want to travel, you can experience global cultural heritage through VR headsets staying at home [16].

### 2.2. Research on the Co-Occurrence Map of VR Headsets Based on Sensor Technology 

Sensor technology is one of the core technologies of VR headsets. VR headsets on the market usually have sound sensors, IMU (Inertial Measurement Unit), distance sensors, Hall sensors, tactile sensors, mechanical sensors, optical sensors, and so on. 

**Figure 1:**
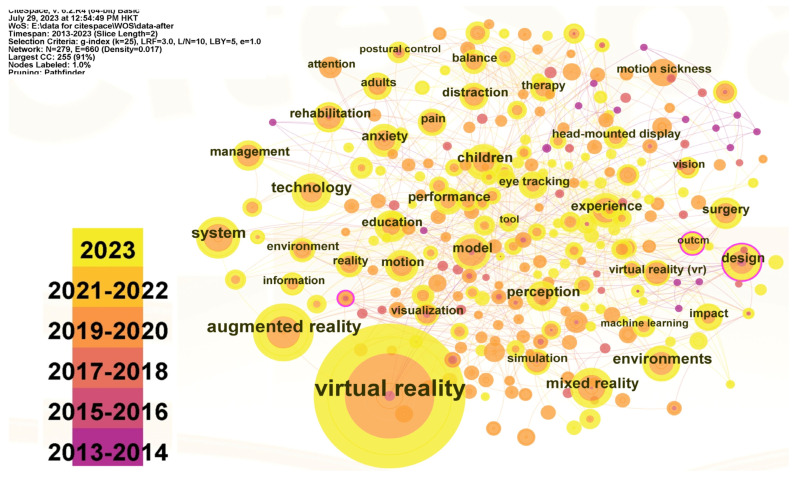
Research on Co-occurrence Map of VR Headsets Based on Sensor Technology.

In this paper, the Citespace software was used to search for papers on the Web of Science over the past ten years, which are listed on SCI, SSCI, and AHCI, and are related to “VR Headset”. A total of 823 related articles were retrieved. The co-occurrence map of sensor technology based on VR headsets was obtained by selecting sensor technology-related keywords from 291 keywords which are classified based on the 823 articles, as shown in Figure 1:

In the co-occurrence map, the higher the frequency of the keywords, the larger the text; and the thicker the ligature between nodes, the stronger the correlation between keywords. Virtual reality in the map is the keyword with the highest frequency. In addition, keywords related to sensor technology are mainly concentrated on vision, eye tracking, walking, movements, and audio. This paper further studies the articles contained in general descriptions such as “design” and “sense”. It is found that in the past 10 years, the research on the sensor of VR headsets mainly focuses on the following three categories:

(1) Distance Sensor: Mainly based on the prediction technology of an inertial measurement unit (IMU) sensor [17], the asynchronous time difference (ATW) [18], and the MEMS ultrasonic sensing technology.
(2) Tactile and Mechanical Sensors: Tactile and mechanical sensors for the Human–Machine Interface (HMI), which include piezoresistive sensors, capacitive sensors, piezoelectric sensors, triboelectric sensors, and wearable skin integrated with tactile and mechanical sensors [7]. It also includes TGI space–time control that can sense hot and cold pain [19].
(3) Optical Sensors: Most of them are optical sensors in head-mounted displays (HMD), and also include a precision task head-mounted projector HMP [20]. In addition, there are few research articles on sound sensors and temperature sensors (mainly used to ensure the stability of Cpu operation) in the field of VR headsets.

### 2.3. Perceived Quality

Olson and Jacoby (1972), the first scholars who proposed perceived quality, defined perceived quality as a method to evaluate product quality [21]. Wheatley (1981) further proposed that perceived quality was not only an evaluation of product quality but also an evaluation of service quality [22]. Zeithaml (1988) then held that perceived quality was about consumers’ perception of a product’s price, quality, and value, and was a judgment of the overall superiority of the product [23,24]. Perceived quality is the evaluation of the overall excellence or advantages of the product; that is, the evaluation of a product [25]. The evaluation results come from comparing actual quality with expected quality. When the product quality is better than the product’s expectations, the perceived quality is good [26]. The evaluation includes the product’s performance and consumers’ overall perception of the overall quality of the product; that is, whether a product or service is superior [27], including a subjective quality evaluation based on product packaging, price, and consumer purchase experience [28].

### 2.4. Perceived Quality Framework

Scholars are currently committed to quantifying the perceived quality of a product or service and its attributes and building its perceived quality framework. Perceived quality elements are the basis of the framework of perceived quality. In terms of how to explore the perceived quality elements of products, according to the clue utilization theory, Olson (1972) proposed quantifying the quality of products or services from internal cues and external cues [21]. The former mainly relies on the physical elements of the products and services; the latter relies on nonphysical elements derived from products or services. Many scholars have proposed different frameworks composed of multiple cue dimensions. Dodds (1991) proposed a five-dimensional (reliability, workmanship, quality, dependability, and durability) perceived quality framework [29]. Robert (1978) held that the most commonly used clues for consumers to choose products are advertising image, individual needs, price, and experience [30]. Zeithaml (2000) proposed six dimensions (usability, multifunctionality, durability, performance, serviceability, and reputation) of the perceived quality framework [23]. Stylidis and Wickman (2019) further put forward the two-dimensional-type theory of perceived quality–technical perceived quality (TPQ) and value-based perceived quality (VPQ), which expands the subject of perceived quality from consumers to producers. TPQ is more inclined to the inherent attributes of products and services. VPQ is more related to the external attributes of products and services such as brand image, product reputation, customer emotional judgment, values, advertising, and after-sales service [31].

### 2.5. Big Data for Online Consumption Reviews

In 2001, the scholar Chatterjee put forward the concept of online review for the first time. Online consumer review refers to the online shopping evaluation of purchased goods or services based on an e-commerce platform after consumers buy products [32]. Online consumption review has the characteristics of wide dissemination, measurable information, and reliable reality and plays an important role and value in many aspects such as consumers and enterprises [33]. Commodity review is an important kind of consumer feedback data, constituting the link between products and consumers, which implies valuable consumer feedback information. How to mine information beneficial to product design, production, and sales from comment data has become the focus of scholars [34].

### 2.6. Perceived Quality Elements 

Perceived quality is a general view of product performance and overall quality; that is, whether the product is superior [27]. These general views are composed of subjective quality evaluations of factors such as product packaging, price, and consumer purchase experience. The perceived quality factors of different products are different, because the product itself can convey different attributes. For analysis on one same product, different researchers adopt different methods and perspectives, which can uncover different perceived quality factors. For example, the perceived quality factors of automobiles that Stylidis analyzed are the qualities of process, aesthetics, physical function, geometry, operating sound, materials, lighting, dynamic and static noise, surface finish, spray paint cover, and so on [35]. The elements excavated by Yang are: modeling design, static perception, operational perception, and dynamic experience [36]. Although there is no research on the perceived quality elements of VR headsets in academic circles, research on consumers’ reviews of VR headsets has emerged. For instance, Amanda believes that a wider perspective can give users a better feeling [37]. The tracking technology of VR headsets can enhance the user’s visual immersion when using the device [38], while Tarr discussed the consumers’ social experiences of VR headsets [39]. However, the comprehensive study of the overall perceived quality elements of VR headsets remains to be studied by scholars.

## 3. Materials and Methods

To obtain the elements of VR headsets’ perceptual quality more comprehensively, this study adopts a two-dimensional perceived quality theory proposed by Stylidis and Wickman (2019) to build a framework of perceived quality [31]. In the research method—a hybrid research metho—a combination of qualitative and quantitative analysis was used. Based on different coexistence goals, different research methods can be embedded in the same research design.

Quantitative analysis:

(1) Organize semi-structured interviews to obtain the preliminary perceived quality elements and framework of a VR headset.
(2) Collect huge numbers of VR headset consumers’ reviews from different websites and obtain the high-frequency words after cleaning the dirty data.
(3) Through the Delphi method, experts can classify the high-frequency words of VR headsets and improve the perceived quality framework.

Qualitative analysis:

(4) Use Likert ten scale to design the importance ranking questionnaire of perceived quality elements.
(5) Distribute, collect, and analyze the questionnaires.
(6) Obtain the final importance ranking of VR headset perceived quality elements.

**Figure 2:**
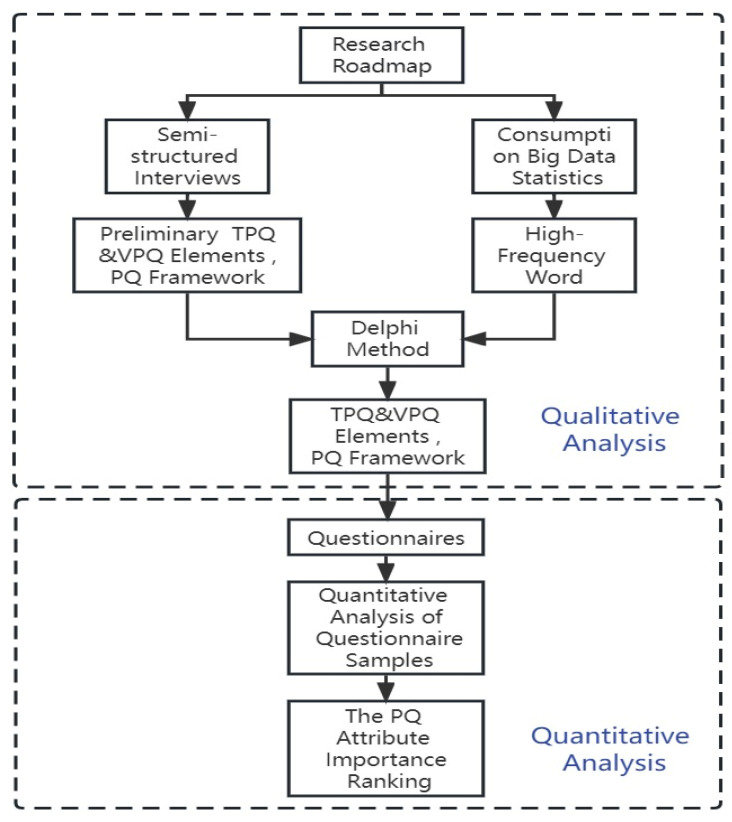
Research roadmap.

Research roadmap is shown in Figure 2:

### 3.1. Semi-Structured Interviews

Found through industry conferences and online forums, 22 professionals were invited to participate in semi-structured interviews. All of them have experienced VR headsets for more than two years. There were more men than women as interviewees, and most of them were young and middle-aged people. About half of the interviewees were industry practitioners and half were consumers. The specific information of the interviewees is shown in Table 1.

**Figure 3:**
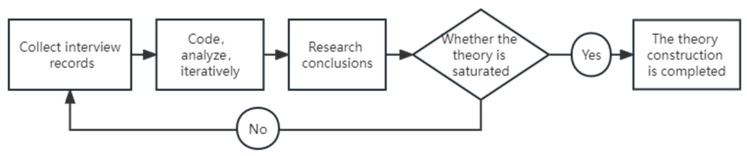
Rooted theory studies flow charts.

The semi-structured interviews, mainly face-to-face include individual interviews and group interviews (no more than five people). The process is as follows:

(1) One day before the interview, the interviewers distributed the same interview guide to the interviewees and informed them that the interview would be conducted according to the content of the interview guide. The respondents were asked to familiarize themselves with the questions in the interview guide.
(2) Based on the Behavioral Event Interview (BEI) developed by Dr. McClelland, this interview collected detailed information on the specific behaviors and psychological activities of the interviewees in representative events by asking a series of questions [40]. Through a comparative analysis of the collected information, key elements can be found. According to BEI, combined with the specific situation of VR headsets, the interviewers developed the following interview guide:
  ① What do you think of the quality of the VR headset?
  ② What memorable events do you have when using the VR headset?
  ③ In your opinion, what aspects of the quality of the VR headset must be guaranteed?
  ④ What needs to be improved in the quality of the VR headset?
  ⑤ What is the most satisfactory thing when you use the VR headset?
  ⑥ What is the most unsatisfactory thing when you use the VR headset?
  ⑦ What other attributes do you think high-quality VR headsets should have in the future?
(3) In the interview, firstly the interviewers introduce the purpose of the interview to the interviewees and explain freedom of speech in that the interview contents are only used for this academic research.
(4) One interviewer talked to the interviewee about the questions in the interview guide, and the other interviewer wrote down the contents of the conversation.
(5) The interview recordings were transcribed into text and the results were proofread.
(6) Grounded theory was used in this study. Grounded theory is a scientific and rigorous qualitative research method, which was first proposed by Glaser and Strauss in 1967 [41]. Through grounded research, the interview records were coded, analyzed, and iteratively synthesized to obtain 11 preliminary perceived quality elements of VR headsets. Rooted theory studies flow charts is shown in Figure 3:
(7) TPQ is more inclined to the inherent attributes of products and services. VPQ is more related to the external attributes of products and services, such as brand image, product reputation, customer emotional judgment, value, advertising, and after-sales service [31]. The organizers divided the initial perceived quality elements of the 11 VR headsets into TPQ and VPQ. The results shown in Table 2 are as follows:
(8) The organizers further classified the initial perceived quality elements of 11 VR headsets, and divided them into three levels, as shown in Table 3:

### 3.2. Big Data Statistics on Consumption

Product comments are significant consumer feedback data. They are the link between products and consumers, implying valuable consumer feedback information [34]. This step focused on mining information beneficial to product design, production, and sales from a large amount of review data.

#### 3.2.1. VR Headsets Consumption Big Data Collection

To understand the elements of the VR headsets’ quality framework more comprehensively and objectively, this study screened consumer reviews of mainstream VR headset products with more than 100 reviews on Taobao, JDOM, Amazon, and other major e-commerce platforms. A total of 56,419 reviews were collected (as of 30 March 2023). Comments covered 15 brands and 35 models, as shown in Table 4:

#### 3.2.2. Big Data Cleaning for Data on VR Headset Consumption

**Figure 4:**
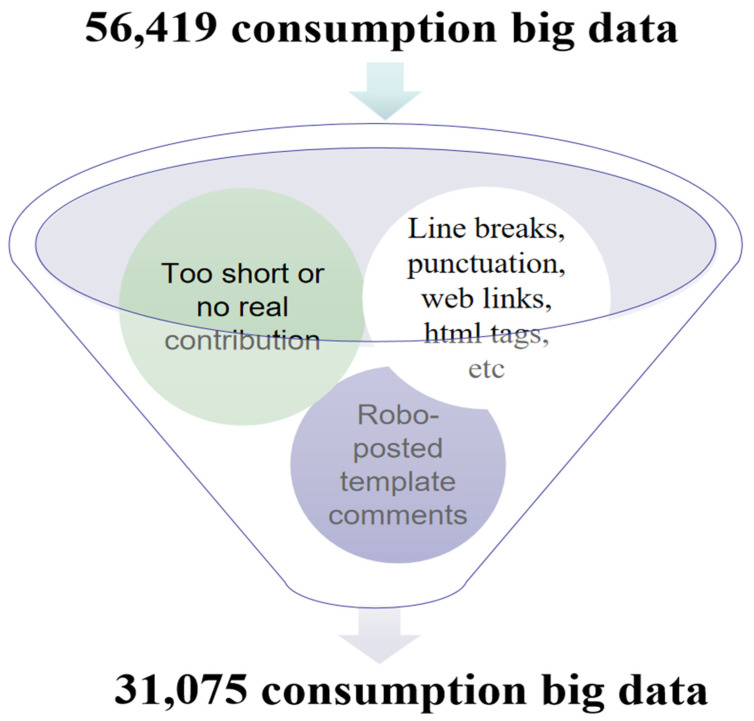
Flow chart showing big data cleaning for VR headset consumption data.

In this study, UTF-8 was used in the coding format. For all crawling data, non-text parts were removed, including new line characters, punctuation marks, web links, HTML tags, etc., and regular expressions were used to filter them. Comments that were too short or did not contribute to the study were deleted from 56,419 samples. In addition, during the process of data collation, the application template comments published by a suspected robot were deleted. After cleaning up the above data, 31,075 of the remaining valid comment data were further processed. Flow chart showing big data cleaning for VR headset consumption data is shown in Figure 4:

#### 3.2.3. Word Frequency Analysis of VR Headsets’ Consumption Big Data Based on TF-IDF

The word frequency can reflect the importance of this word in the text and present the content of the text provider’s attention [42]. By extracting those high-frequency words from the consumers’ reviews of VR headsets, we can obtain the focus of consumers on VR headsets, which can deeply explore the perceived quality framework of VR headsets. 

**Figure 5:**
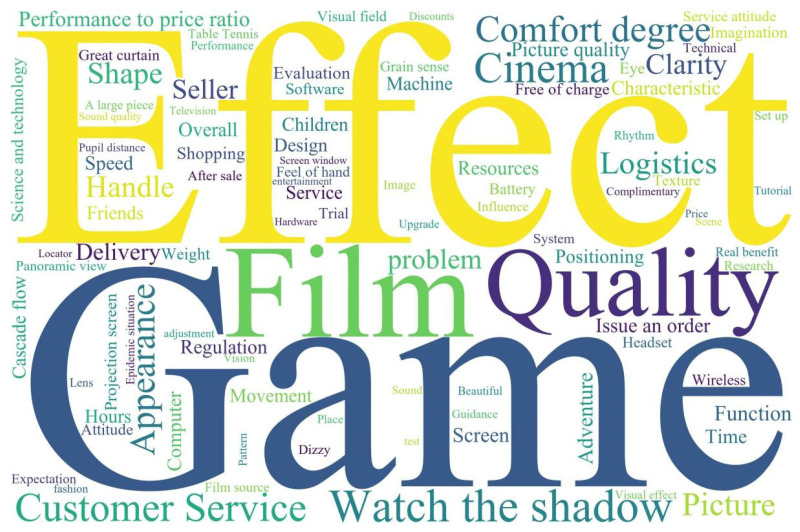
Cloud diagram showing high-frequency words.

Term frequency–inverse document frequency (TF-IDF) is a common statistical method. TF is the frequency of words in the traditional sense, meaning the frequency of a word in the text. IDF is “inverse text frequency”, which means that when a word appears very frequently in all texts, such as a preposition or a modal auxiliary, it may not be as important as some less frequent words. With Jieba participle software data segmentation and, subsequently, Rost software for word segmentation of data statistics, the study obtained consumers’ assessments of VR headsets through high-frequency words. Cloud diagram showing high-frequency words is shown in Figure 5:

### 3.3. Classification of High-frequency Words by the Delphi Method

By screening, cleaning, and merging high-frequency words, this study produced a high-frequency vocabulary list of the top 100 ranking words related to VR headset consumption. A total of 22 interviewees who had previously participated in semi-structured interviews were invited to form an expert group and asked to evaluate these high-frequency words and categorize them into the preliminary perceptual quality framework. High-frequency words related to VR headset use are shown in Table 5:

The organizer collected and summarized the classification results of all interviewees in the first round and then sent them to each interviewee, asking them to compare their different opinions with others in the first round of evaluation, modify their opinions and judgments, and form the results of the second round of evaluation. The organizer then collected and summarized the results of the second round of evaluation of all interviewees and sent them to each interviewee, asking them to compare their different opinions with others in the second round of evaluation, modify their opinions and judgments, and form the results of the third round of evaluation. Finally, after three rounds of assessment, none of the interviewees changed their opinions. Organizers summarized the results of the third round of evaluation, and they integrated and unified the naming of the results, classifying words such as “friends” and “children” into the same category; however, some respondents used words such as “socializing”, while others used words such as “social relation”. The organizer returned to the original consumption review information, combed the content when referring to friends, children, and other words, and found that most of these words were mentioned enough times to evaluate feelings towards VR headsets with friends and children. Therefore, the organizer determined that this category would be labeled as “socializing”. After the final evaluation results were integrated with Table 2 and Table 3, a list of the perceived quality elements (Table 6) and the VR headsets’ perceived quality framework (Table 7) screened by industry experts and consumers was obtained. Table 6 added five new elements: Multifunctionality, Pupillary distance regulation, Equipment weight, Material, and Socializing. There were 6 items in Level I of Table 7, and Compatibility was added. There were 12 items in Level II, newly including the Myopia application, Wear suitable, Software durability, and Interaction.

### 3.4. PQAIR of VR Headsets

Bi (2004) proposed a set of processes according to the order of many elements that affect the formation and degree of customer-perceived quality: firstly, study the significant stages and elements; then, determine the critical elements of customer-perceived quality from the customer perspective [43]. This study ranked the importance of perceived quality attributes of VR headsets by analyzing the questionnaire results.

#### 3.4.1. Questionnaire Design

The Likert scale is a compound measurement developed by American social psychologist Likert in 1932. It attempts to improve the level of measurement in social research by using standardized answer classifications in a questionnaire survey and to determine the relative intensity of different items. The Richter scale is a single stimulus–response model (single stimulus–response format). Under the single stimulus–response model, subjects need to judge whether the topic description agrees with their characteristics according to the description of topic and then choose the degree of conformity with themselves on the Richter scale, which usually ranges from very disagreeable to very agreeable.

To ensure the smooth progress of the survey, during the process of questionnaire design, the interviewees’ answer experience was fully considered during the question design process. Because there were many items in the main part of the questionnaire, to ensure the reliability of the analysis and to obtain better results, a level 10 Rickett scale (10-point Likert scale) was used to indicate the extent to which investigators pay attention to the project (1 was the lowest and 10 was the most significant).

#### 3.4.2. Questionnaire Distribution and Collection

The questionnaire was mainly administered offline and online and was completed by VR industry practitioners, VR enthusiasts, and consumers. The interviewees who participated in the survey had either purchased or learned about VR products. A total of 383 questionnaires were distributed. After further screening, 340 valid questionnaires were obtained, with an effective rate of 88.77%. The demographic data of the respondents to the questionnaire are presented in Table 8 and Table 9.

#### 3.4.3. Quantitative Analysis of Questionnaire Samples

Cronbach’s α coefficient is one of the most commonly used reliability indicators, indicating the consistency of scores between items in a scale. The Cronbach’s α coefficient of the questionnaire took advantage of the scale module of the SPSS 11.5 software. The SPSS reliability analysis results are presented in Table 10.

From the reliability statistics, it can be concluded that the analysis results were acceptable.

The modified correlation coefficient (CITC) was used in the reliability analysis to purify the measurement indicators, detect the impact of each element on the overall reliability, and find elements with problems in the questionnaire design. Then, the study used Cronbach’s α coefficient to test the reliability of the questionnaire again. When using CITC-modified reliability screening items, the following criteria were used to identify problematic elements:

The revised item correlation coefficient CITC (the correlation coefficient between each item score and the remaining item score) was less than 0.3; if the item was deleted, the α value, indicating overall reliability, increased.

CITC analysis was performed on all 16 items. The reliability analysis results for all indicators are presented in Table 11.

From the analysis results of the CITC index, the CITC value of most items was higher than the standard value of 0.3. Among them, the CITC value of pupillary distance regulation was 0.200, and the CITC value of hardware compatibility was 0.064. The CITC values of the two items were less than the standard value of 0.3 and, after deleting these two items, the overall Cronbach’s α value improved. According to the CITC deletion principle, this indicator can be deleted. After deletion, the results for Cronbach’s α are displayed in Table 12.

After analyzing the two items, pupillary distance regulation and hardware compatibility were deleted. Their CITC values were lower than the certified value. This can be explained by the mean variance of 340 valid questionnaires of these two items, as shown in Table 13.

As for pupillary distance regulation, the interviewees disagreed, and they judged the importance of this item based on the basis of whether they were myopic. As a result, interviewees without myopia thought this item was not significant, while those with myopia thought it was significant. The survey results are subjective, and this item is not suitable for questionnaire design.

For hardware compatibility, interviewees also showed large differences. We speculate that the reason may be that the condition of the hardware owned by interviewees was quite different; e.g., the hardware configuration of the host was different. Some hosts are compatible with each other. These kind of interviewees are not troubled by poor compatibility.

On the premise of reliability, the study further used the KMO test and Bartlett spherical test to analyze the structural validity of the questionnaire samples. The results are as presented in Table 14.

The KMO test is used to test the partial correlation of variable construction. The closer the KMO test value is to 1, the better the structural validity is. If the KMO test value is less than 0.5, the structural validity of the questionnaire sample is considered unacceptable. The KMO test value of the questionnaire sample after deleting the question item was 0.861, which indicated that the selected structural sample was suitable for factor analysis.

If the result of the Bartlett spherical test is less than 0.005, the spherical hypothesis is rejected, which indicates that there is a strong correlation between variables. The spherical test result of this questionnaire sample was 0; thus, we can infer that the questionnaire possessed good structural validity.

With the results of the Cronbach’s α coefficient, KMO test, and Bartlett spherical test, we concluded that the design and results of the hypothetical variables in the questionnaire were consistent and credible.

## 4. Results

According to the scores for the importance of each index in the questionnaire sample, we used the mean analysis method of perceived quality attribute importance ranking (PQAIR) to rank the VR headsets from high to low, as shown in Table 15.

The PQAIR of the studied VR headsets can be obtained through the study of questionnaire samples. The average value of each element represents the average score of the element in the whole questionnaire sample. The higher the score, the higher the importance of attributes. Variance reflects the difference between each sample and the mean value. The higher the variance is, the greater the fluctuation of the element in the whole questionnaire sample; that is to say, the greater the difference between different interviewees for the element. The mean variance statistics are based on all 340 valid questionnaires.

## 5. Discussion

In the PQAIR of VR headsets, somatosensory immersion is the most important factor for manufacturers and consumers, because the VR headsets are essentially a system designed to improve immersion by installing wide field displays within the user’s line of sight [44]. Somatosensory dizziness ranks sixth in importance; somatosensory dizziness refers to the limitation of VR headsets in terms of possible network disease, which may lead to headaches, nausea, and other symptoms. There are several terms for this phenomenon: motion sickness, simulator disease, and virtual reality disease [45]. When we compiled the big data statistics on the e-commerce website, we found that users frequently used words such as “immersive experience” when making positive comments while, when making negative comments, they often described their dizziness and discomfort after using VR headsets.

In addition to the somatosensory attributes, the line-of-sight attribute is an important attribute that affects the quality of perception. The elements following the line-of-sight attribute, in terms of importance, included tracking accuracy (third), resolution (fifth), refresh rate (seventh), and visual angle (ninth). Tracking accuracy in VR headsets usually means that the system can accurately capture the trajectory of the consumers’ heads. In many VR headset game usage scenarios, the movement of the head determines the direction and location in which the consumers can see the virtual scene. If the tracking accuracy is low and there is a mismatch between vision and the vestibule system, users who wear VR headsets will feel dizzy [46]. The difference between the motion time captured by the consumers through the sensor and when the image appears on the headsets makes the motion-to-photon (MTP) and offset feel stronger. The long MTP delay is a significant reason for why consumers feel sick and carsick [47]. The higher the resolution of the product, the sharper, more detailed, and more realistic the image displays. The refresh rate is also known as the frame rate (frames per second), which refers to the number of refresh times of images on the screen. The refresh rate of the mainstream VR headsets in the current market is generally between 80–120 Hz. The higher the resolution, the smoother and more realistic the virtual reality experience. Whether watching movies or playing games, resolution and refresh rate are significant guarantees for providing consumers with a high-quality visual experience. Visual angle determines the angle of the visible area when the consumer uses the VR headsets; it determines the range of images that the consumer can see. The wider the image range, the stronger the consumer’s immersion. One eye has a field of view of about 160 degrees (lateral) times 130 degrees (vertical). The combined binocular field of view is about 200 degrees (laterally) multiplied by 130 degrees (vertically); the overlap is 120 degrees. The perspective of the VR headsets on the market is generally between 80 degrees and 120 degree, and the visual angle of some products, such as the Pimax Vision 8K, can even reach 200 degrees. Tethered VR headsets and all-in-one VR headsets, as wearable devices, have a small optical display in front of at least one wearer’s eyes [48]. Visual experience interaction is the most basic human–computer interaction experience between VR headsets and consumers. Therefore, to improve the experience of human–computer interaction, VR headsets must pay attention to consumers’ positive sight experience when using VR headsets.

From the perspective of sociality, including brand reputation (fourth in rank), after-sale service (ranked eight), and socializing (ranked thirteenth), the brand of VR headsets has a positive impact on product value creation [49], and a good brand reputation can significantly enhance the perceived value of VR headsets. For this kind of technology product, in addition to the purchase evaluation of the product on electronic commerce websites, consumers can find commodity evaluations and consumer experiences on domestic social media platforms such as WeChat and Weibo as well as on word-of-mouth websites such as Meituan Dianping. In addition to offline VR headset exhibitions, video sites such as Bilibili and YouTube are significant channels for accumulating brand reputation. The impact of the after-sale service on consumers is mainly reflected in the warranty strategies provided for different products. When consumers choose foreign brands, the impact of the after-sale service is especially more notable. Within the same country, different brands often have little difference in after-sales service strategies. The elements of VR headsets in socializing are mainly focused on VR social networks and the VR online game scene. Currently, VR headsets are combined with social network services and consumers can interact in VR-based social network services (SNS) as avatars [50]. Furthermore, some VR-based chat products have been created, but the majority of interviewees in this study had not learned about these products; most of the knowledge surrounding socializing focused on VR online game scenarios. In the VR online game scene, the socializing element has not received a great deal of attention. On the one hand, the interviewees think that the mainstream VR headsets in the current market are more mature in the network online, and the online speed and online delay usually depend on the network situation of the consumers, rather than the VR headsets themselves. On the other hand, in VR online games, socializing depends more on the optimization of the game itself than on the VR device. In summary, with regard to socializing, products with good brand reputations often have better perceived quality, which is also the element that consumers pay more attention to when choosing VR products; consumers pay more attention to products with a warranty period compared to products without one. Products that can provide stable quality assurance for some time have more advantages. Human–computer interaction is highly correlated with service experience [51]. The service experience before and after the use of VR headsets also affects the experience of the consumer’s human–computer interaction. For socializing, many people use VR headsets for social interaction, which increases the scene of human–computer interaction while increasing the fun of the use of products, but consumers generally believe that socializing depends more on the network environment and VR multi-person interaction software optimization, rather than VR headsets themselves, with interviewees believing that different VR headsets make little difference in socializing.

Regarding durability, this paper studied durable software and hardware durability, in which durable software (ranked second) mainly refers to VR resource quality, while hardware durability includes battery life (ranked eleventh) and material (ranked twelfth). In this study, VR resource quality ranked second and was a significant part of the perceived quality of VR headsets. The human–computer interaction between consumers and VR headsets is concentrated in three major content areas: VR videos, VR games, and VR industry applications. Different brands usually have their resources exclusively in VR videos and VR games. When consumers choose different brands of VR headsets, they tend to pay more attention to the exclusive VR resources of the brand, which are only available in the corresponding brand’s App Store. These exclusive VR resources tend to have high game quality, and players who like these games must purchase the corresponding VR headset to play these exclusive games, which are not carried by the app stores for other brands. Experiencing the unique content brought by VR technology is also the purpose of interpersonal interaction between consumers and VR headsets. VR videos and VR games are the human–computer interaction scenes most frequently used by consumers. However, the VR industry is still in the stage of rapid development. Industries such as tourism and education, sports entertainment, real estate, medical treatment, etc., have now begun to try applying VR technology, e.g., Stewart Birrell (2022) using a tethered VR headset device to allow participants to use controller navigation in the virtual environment of urban airports [52]. Virtual reality technology is now used to treat sensorimotor disorders in medical environments [53]. These industry applications represent the future development direction of VR headsets, which will become more comfortable and more suitable for multiple environments. The aspects of hardware durability, battery life, and material had some influence on the perceived quality of the interviewees. The battery life of mainstream VR headsets is generally about three hours while, in general, the interviewees usually use VR headsets continuously for between half an hour and one hour.

More than half of the interviewees said they had a more obvious sense of fatigue after half an hour of continuous use, and after more than one hour of continuous use, there was often a certain degree of discomfort; thus, three hours of battery life is usually adequate for consumers. The influence of material on durability is more focused on the performance of product features such as anti-fall, being waterproof, etc. To reduce the weight of VR headsets, most VR headsets use plastic fuselage, which also poses a higher challenge to the anti-fall ability of the product. However, due to the use of the environment, many interviewees also said that the material is not an element they focus on. In this study, interviewees paid more attention to durable software than the impact of hardware durability on perceived value. The perceived quality element of compatibility equipment weight only ranked 10th but was indicated to improve comfort. Sai Akhil Penumudi (2020) demonstrated that improper neck flexion torque and musculoskeletal load will cause discomfort when consumers use VR equipment [54]. Neck joint torque is significantly affected by mass and compatibility, which increases with the weight of VR headsets [55]. Therefore, equipment weight overload will cause musculoskeletal discomfort for consumers, affecting their personal interaction experience. In the study, interviewees generally said that the impact of equipment weight on compatibility was more focused on the ergonomic design of products. VR headsets with good equipment weight tend to be more comfortable to wear, and longer use does not easily produce fatigue; on the other hand, products with poor equipment weight are more likely to be uncomfortable to wear.

Finally, the perceived quality element of multifunctionality in usability ranked last. The study found that almost all interviewees had some knowledge of PC and mobile operations, which enabled them to be started faster when using VR headsets. At the same time, the UI interface of most of the operating systems used in VR headsets is flattened, which makes both the menu bar and icon display more intuitive, making it easy for consumers to find most of the features they want. Although some new VR industry applications or games still have a learning threshold for consumers, multifunctionality also creates more possibilities for VR headset consumers. For example, Woojoo Kim (2022) proposed VR headsets without additional equipment to satisfy consumers who wish to obtain experiential effects in virtual reality text input [56]. The development of multifunctionality in VR headsets is key to their future application scenarios and potential, and interviewees generally have a high degree of tolerance for it. Combined with the conclusions of this study, it can be further seen that in the field of commercial VR headsets, immersion, tracking accuracy, dizziness, and other elements related to distance sensing technology are more concern by consumers, while in optical sensing technology, consumers pay more attention to resolution, refresh rate, and visual angle. For tactile and mechanical sensors, consumers have not paid much attention, which means that the effect of technology investment in this area on promoting consumer purchases is not significant.

## 6. Conclusions

This paper discusses the importance ranking of the perceived quality factors of VR headsets, aiming to discuss and verify the rationality of the optimization of various sensing technologies from the perspective of the market and consumers. Different from most previous research perspectives, this study innovatively combines TPQ, VPQ, and big data statistical analysis technology, not only taking the views of VR headset industry experts and designers into consideration, but also considering the consumers’ attitudes towards the VR headsets to study the perceived quality elements. Through this study, we obtained the importance ranking of different perceived quality elements. We concluded the importance ranks of different perceived quality factors in this research. For VR headset products, these quality factors include not only the future optimization direction of sensing technology but also the improvement suggestions about marketing, after-sales service, and so on. This is of great significance for VR headset manufacturers and designers to create market-competitive VR headsets.

In future research, we will continue to focus on the perceived quality of wearable devices, not only the VR headsets. There are two research directions in the future: one is to expand the research of consumer big data to the global e-commerce platform, strive to obtain a wider range of consumers’ feedback on VR headsets, then analyze the consumer perception through machine learning algorithms; the other is to further subdivide the user groups and usage scenarios for the user’s optimization suggestions for distance sensing technology and optical sensing technology, and study the sensitivity and acceptable range of specific users for various sensors in specific scenarios.

## Figures and Tables

**Table 1:** Basic information of interviewees in semi-structured interviews.

Characteristics	Grouping	Number of People	Percentage
Gender	Male	17	77.27%
	Female	5	22.73%
Age	18–24 years old	3	13.64%
	25–35 years old	11	50.00%
	36–45 years old	6	27.27%
	46–55 years old	2	9.09%
Personnel Type	Industry expert	5	22.73%
	Senior VR enthusiasts	4	18.18%
	Internet industry experts	3	13.64%
	VR headsets consumers	10	45.45%

**Table 2:** Preliminary perceived quality elements of VR headsets.

Elements	TPQ	Elements	VPQ
1	Visual angle	1	Immersion
2	Resolution	2	Dizziness
3	Refresh rate	3	After-sales service
4	Hardware compatibility	4	Brand reputation
5	VR resource quality		
6	Tracking accuracy		
7	Battery life		

**Table 3:** Preliminary perceived quality framework of VR headsets.

Level I	Level II	Level III
Sight	Picture clarity	Visual angle
		Resolution
	Picture fluency	Refresh rate
		Tracking accuracy
Somatosensory	Positive feeling	Immersion
	Negative feelings	Dizziness
Durability	Hardware durability	Battery life
	Durable software	VR resource quality
Usability	Hardware usability	Hardware compatibility
Sociality	Goodwill	Brand reputation
	Service	After-sales service

**Table 4:** Brands and models of VR headsets.

No.	Brand	Product Model
1	DP	DPVR E3
2		DPVR E4
3	GOOVIS	GOOVIS G2-X
4		GOOVIS G3
5		GOOVIS Lite
6		GOOVIS Pro-X
7	HP	HP Reverb G2
8	HTC	HTC Vive CE
9		HTC Vive Cosmos
10		HTC Vive Focus3
11		HTC Vive Pro 1.0
12		HTC Vive Pro 2.0
13		HTC ViveFlow
14	HUAWEI	HUAWEI VR GlASS
15	iQIYI	iQIYI 2Pro
16		iQIYI 4KVR
17		iQIYI Dream Pro
18	Microsoft	Microsoft HoloLens 2
19	NOLO	NOLO X1 6DoF
20	Oculus/Meta	Oculus/Meta Quest 2
21		Oculus/Meta Quest 2 Pro
22		Oculus/Meta Rift S
23	Pico	Pico 4
24		Pico 4 Pro
25		Pico Neo2 Lite
26		Pico Neo3
27	Pimax	Pimax 8KX DMAS
28		Pimax Vision 8K Plus
29		Pimax Vision 8KX
30	SAMSUNG	SAMSUNG Gear 4KVR
31	Skyworth	Skyworth-S801
32		Skyworth-S802
33		Skyworth-V901
34	Valve	Valve Index1.0
35		Valve Index2.0

**Table 5:** High-frequency words related to VR headset use.

No.	Words	Frequency	No.	Words	Frequency
1	Game	7787	51	Trial	429
2	Effect	7571	52	Great curtain	428
3	Film	4209	53	Headset	424
4	Quality	3930	54	Wireless	422
5	Watch the shadow	3829	55	Texture	408
6	Cinema	3271	56	Visual field	400
7	Customer Service	2909	57	Service attitude	397
8	Comfort degree	2397	58	Grain sense	382
9	Appearance	2210	59	Feel of hand	380
10	Picture	1836	60	System	354
11	Logistics	1729	61	Panoramic view	349
12	Handle	1609	62	After sale	334
13	Shape	1608	63	Influence	324
14	Clarity	1472	64	Expectation	321
15	problem	1369	65	Technical	318
16	Seller	1366	66	Research	318
17	Delivery	959	67	Film source	313
18	Performance to price ratio	922	68	Table Tennis	301
19	Adventure	910	69	Dizzy	296
20	Function	903	70	Set up	293
21	Screen	837	71	Real benefit	286
22	Issue an order	795	72	Image	275
23	Picture quality	767	73	A large piece	272
24	Computer	754	74	Rhythm	266
25	Regulation	753	75	Lens	259
26	Resources	746	76	Performance	256
27	Time	745	77	Beautiful	247
28	Characteristic	745	78	Guidance	245
29	Speed	742	79	Epidemic situation	242
30	Overall	741	80	Place	242
31	Positioning	709	81	Visual effect	238
32	Service	688	82	Complimentary	231
33	Movement	684	83	Tutorial	231
34	Friends	678	84	Sound	229
35	Hours	678	85	Upgrade	225
36	Machine	675	86	Vision	224
37	Children	666	87	Pattern	223
38	Cascade flow	658	88	fashion	221
39	Design	655	89	Pupil distance	220
40	Evaluation	563	90	adjustment	207
41	Shopping	562	91	Television	202
42	Science and technology	536	92	Price	200
43	Projection screen	521	93	Discounts	197
44	Weight	500	94	Scene	196
45	Free of charge	485	95	test	194
46	Software	485	96	Sound quality	192
47	Eye	478	97	entertainment	187
48	Imagination	455	98	Screen window	186
49	Attitude	453	99	Hardware	185
50	Battery	439	100	Locator	184

**Table 6:** Perceived quality elements of VR headsets.

Elements	TPQ	Elements	VPQ
1	Visual angle	1	Immersion
2	Resolution	2	Dizziness
3	Refresh rate	3	After-sales service
4	Hardware compatibility	4	Brand reputation
5	Multifunctionality	5	VR resource quality
6	Tracking accuracy	6	Socializing
7	Pupillary distance regulation		
8	Battery life		
9	Equipment weight		
10	Material		

**Table 7:** Perceived quality framework of VR headsets.

Level I	Level II	Level III
Sight	Picture clarity	Visual angle
		Resolution
	Picture fluency	Refresh rate
		Tracking accuracy
Somatosensory	Positive feeling	Immersion
	Negative feeling	Dizziness
Compatibility	Myopia application	Pupillary distance regulation
	Wear suitable	Equipment weight
Durability	Hardware durability	Material
		Battery life
	Software durability	VR resource quality
Usability	Hardware usability	Hardware compatibility
		Multifunctionality
Sociality	Goodwill	Brand reputation
	Service	After-sale service
	Interaction	Socializing

**Table 8:** PQAIR of VR Headsets.

Visual angle	1	2	3	4	5	6	7	8	9	10
Resolution	1	2	3	4	5	6	7	8	9	10
Refresh rate	1	2	3	4	5	6	7	8	9	10
Hardware compatibility	1	2	3	4	5	6	7	8	9	10
VR resource quality	1	2	3	4	5	6	7	8	9	10
Multifunctionality	1	2	3	4	5	6	7	8	9	10
Tracking accuracy	1	2	3	4	5	6	7	8	9	10
Pupillary distance regulation	1	2	3	4	5	6	7	8	9	10
Battery life	1	2	3	4	5	6	7	8	9	10
Equipment weight	1	2	3	4	5	6	7	8	9	10
Material	1	2	3	4	5	6	7	8	9	10
Socializing	1	2	3	4	5	6	7	8	9	10
Immersion	1	2	3	4	5	6	7	8	9	10
Dizziness	1	2	3	4	5	6	7	8	9	10
Brand reputation	1	2	3	4	5	6	7	8	9	10
After-sale service	1	2	3	4	5	6	7	8	9	10

**Table 9:** Demographics of the PQAIR of VR headsets questionnaire.

Characteristic	Grouping	Frequency	Percentage
Gender	Male	234	68.82%
	Female	106	31.18%
Age	Under 18 years old	28	8.24%
	18–24 years old	91	26.76%
	25–35 years old	134	39.41%
	36–45 years old	69	20.29%
	46–55 years old	18	5.29%
Monthly income (RMB)	Less than 500 dollars	32	9.41%
	500–715 dollars	43	12.65%
	715–1000 dollars	79	23.24%
	1000–1285 dollars	87	25.59%
	1285 dollars and above	99	29.12%
Education level	High school or below	29	8.53%
	Junior college	95	27.94%
	Undergraduate	166	48.82%
	Graduate students and above	50	14.71%

**Table 10:** The overall reliability analysis of the questionnaire.

Reliability Statistics	
Cronbach’s Alpha	N of Items
0.844	16

**Table 11:** Analysis of the items’ CITC reliability.

Item-Total Statistics				
	Scale Mean if Item Deleted	Scale Variance if Item Deleted	Corrected Item-Total Correlation	Cronbach’s Alpha if Item Deleted
Visual_angle	87.83	169.231	0.333	0.841
Pupillary_distance_regulation	88.78	168.279	0.2	0.854
Resources	86.24	155.422	0.724	0.822
Brand_reputation	87.22	155.264	0.784	0.82
Resolution	87.54	164.119	0.413	0.837
Immersion	85.43	159.414	0.676	0.825
After_sales_service	87.66	154.085	0.607	0.826
Material	88.71	152.648	0.647	0.824
Multifunctionality	89.44	165.22	0.407	0.838
Refresh_rate	87.64	160.029	0.437	0.836
Tracking_accuracy	86.94	163.252	0.406	0.838
Equipment_weight	87.84	167.182	0.391	0.838
Dizziness	87.59	156.543	0.615	0.826
Baterry_life	88.08	162.569	0.472	0.834
Socializing	89.02	163.793	0.418	0.837
Hardware_compatibility	88.12	177.365	0.064	0.856

**Table 12:** Revised overall reliability statistics.

Reliability Statistics	
Cronbach’s Alpha	N of Items
0.868	14

**Table 13:** Mean and variance statistics of deleted items.

Item Statistics			
	Mean	Std. Deviation	Number
Pupillary_distance_regulation	4.83	1.995	340
Hardware_compatibility	5.48	1.588	340

**Table 14:** KMO test and Bartlett spherical test.

KMO and Bartlett’s Test		
Kaiser–Meyer–Olkin Measure of Sampling Adequacy.		0.861
Bartlett’s Test of Sphericity	Approx. Chi-Square	2705.056
	Df	91
	Sig.	0

**Table 15:** PQAIR of VR headsets (after removing distortion elements).

Rank	Element	Attribute	Mean Value	Variance	N
1	Immersion	Somatosensory	8.18	1.264	340
2	VR resource quality	Durability	7.36	1.396	340
3	Tracking accuracy	Sight	6.67	1.612	340
4	Brand reputation	Sociality	6.39	1.311	340
5	Resolution	Sight	6.06	1.526	340
6	Dizziness	Somatosensory	6.01	1.538	340
7	Refresh rate	Sight	5.96	1.76	340
8	After-sale service	Sociality	5.95	1.7	340
9	Visual angle	Sight	5.77	1.337	340
10	Equipment weight	Compatibility	5.77	1.344	340
11	Battery life	Durability	5.53	1.482	340
12	Material	Durability	4.89	1.693	340
13	Socializing	Sociality	4.58	1.537	340
14	Multifunctionality	Usability	4.17	1.482	340

## Data Availability

The data presented in this study are available on request from the corresponding author.
